# Incidence rates of classical Kaposi's sarcoma and multiple myeloma do not correlate.

**DOI:** 10.1038/bjc.1998.509

**Published:** 1998-08

**Authors:** H. Hjalgrim, M. Frisch, M. Melbye

**Affiliations:** Department of Epidemiology Research, Danish Epidemiology Science Centre, Statens Serum Institut, Copenhagen.

## Abstract

We compared population-based incidence rates for classical Kaposi's sarcoma and multiple myeloma. Neither for men (Spearman's rank correlation coefficient (r) = 0.01, P = 0.97, 13 pairs) nor for women (r = 0.24, P = 0.42, 13 pairs) did the incidences of the two conditions correlate. This absence of correlation does not support the hypothesis that Kaposi's sarcoma and multiple myeloma share a common aetiology, such as HHV-8.


British Journal of Cancer (1998) 78(3). 419–420
© 1998 Cancer Research Campaign

Short communication

### Incidence rates of classical Kaposi's sarcoma and multiple myeloma do not correlate

**H Hjalgrim, M Frisch and M Melbye**

Department of Epidemiology Research. Danish Epidemiology Science Centre. Statens Serum Institut. 5 Artillerivej. DK-2300 Copenhagen. Denmark

**Summary** We compared population-based incidence rates for classical Kaposi's sarcoma and multiple myeloma. Neither for men (Spearman's rank correlation coefficient ($r$) = 0.01, $P$ = 0.97, 13 pairs) nor for women ($r$ = 0.24, $P$ = 0.42, 13 pairs) did the incidences of the two conditions correlate. This absence of correlation does not support the hypothesis that Kaposi's sarcoma and multiple myeloma share a common aetiology, such as HHV-8.

**Keywords:** Kaposi's sarcoma; multiple myeloma; human herpesvirus-8

Recently, a molecular study of bone marrow tissue from patients with multiple myeloma (MM) suggested that this condition should be added to the list of diseases presumably caused by human herpesvirus-8 (HHV-8) (Rettig et al. 1997*a*). Previous follow-up studies of patients with Kaposi's sarcoma (KS) have provided little evidence to suggest that these patients are at increased risk of MM (Grulich et al. 1992; Biggar et al. 1994; Hjalgrim et al. 1997). Estimates of the prevalence of HHV-8 currently exist for a few populations only, and accordingly it is not possible to relate such measures to MM occurrence. However, reliable incidence rates of classical KS have become available for many geographical regions, demonstrating a substantial variation (Hjalgrim et al. 1998). Studies of different groups at increased risk of KS, e.g. HIV-infected patients, have indicated that prevalence of HHV-8 correlates with KS occurrence (Gao et al. 1996; Simpson et al. 1996). This suggests that disease occurrence may reflect virus prevalence. We therefore hypothesized that if HHV-8 were causally involved in the pathogenesis of MM, incidence rates of MM and classical KS would correlate.

### MATERIAL AND METHODS

Crude incidence rates for MM in different populations for the period 1978–82 were obtained from *Cancer Incidence in Five Continents* (Muir et al. 1987) (Figure 1). These were paired with corresponding rates of classical KS (crude or standardized to local populations) available in the literature or from recent studies of KS in the Scandinavian countries (Figure 1). Correlation between the rates for the two conditions was tested by means of Spearman's rank test.

### RESULTS

A total of 13 pairs of incidence rates for KS and MM were identified for both men and women (Figure 1). The occurrences of KS and MM did not correlate. This applied to analyses of paired data (men and women together) ($r$ = 0.15, $P$ = 0.46), as well as to separate analyses for men ($r$ = 0.01, $P$ = 0.97) and women ($r$ = 0.24, $P$ = 0.42).

**Figure 1** Crude incidence rates of multiple myeloma by corresponding rates for classical Kaposi's sarcoma. Periods and sources for Kaposi's sarcoma incidence rates: UK 1971–80 (Grulich et al. 1992): Australia 1972–82 (Kaldor et al. 1994): USA and Puerto Rico 1973–79 (Biggar et al. 1984): Ragusa, Italy, 1981–84, Parma, Italy, 1978–84 and Varese, Italy, 1976–84 (Geddes et al. 1994): Iceland, 1975–79 (Hjalgrim et al. 1998). Crude rates for Denmark, Sweden, Norway and Finland, all 1978–79, have been extracted from (Hjalgrim et al. 1996). Data from the Swiss canton of Vaud were included in the analyses, but do not appear in the figure (Levi et al. 1993)

*Received 17 December 1997*
*Revised 27 January 1998*
*Accepted 28 January 1998*

*Correspondence to:* H Hjalgrim

### DISCUSSION

Since the initial description of HHV-8 in 1994 (Chang et al. 1994), there has been an intense search for conditions potentially caused by the virus. Beside KS, there is, at present, evidence to suggest that HHV-8 is involved in the pathogenesis of Castleman's disease (Dupin et al. 1995) and primary effusion lymphomas (Cesarman et al. 1995). Most recently, it has been suggested that HHV-8 may also be involved in the development of MM (Brousset et al. 1997; Rettig et al. 1997*a* and *b*; Said et al. 1997; Tisdale et al. 1997). The present study, however, finds that population-based incidence rates of classical KS and MM do not correlate. This result is in accordance with serological and molecular studies of patients with MM demonstrating no unusual prevalence of HHV-8 in this group of patients (Cesarman et al. 1995; Pastore et al. 1995; Cook et al. 1997; MacKenzie et al. 1997; Marcelin et al. 1997; Masood et al. 1997; Parravicini et al. 1997; Whitby et al. 1997). Similarly, follow-up studies of patients with classical or AIDS-related KS have not provided data indicative of an increased risk of MM among such patients (Grulich et al. 1992; Biggar et al. 1994; Hjalgrim et al. 1997).

The present study has certain limitations. For instance, some geographical variation in completeness of registration of MM and KS may exist. Moreover, while only crude incidence rates of MM are included in the analyses, a few of the KS incidence rates were standardized to local populations, as of 1970 (USA and Iceland). It is unlikely, however, that this has had any influence on the apparent absence of correlation between KS and MM incidence rates.

We therefore conclude that the hypothesis that KS and MM share a common aetiology, such as HHV-8, is not supported by currently available epidemiological evidence. However, we cannot rule out the possibility that different strains of HHV-8 may cause different diseases, e.g. KS and MM, as has been suggested recently (Luppi et al. 1997).

### REFERENCES

Biggar RJ, Horm J, Fraumeni JF Jr, Greene MH and Goedert JJ (1984) Incidence of Kaposi's sarcoma and mycosis fungoides in the United States including Puerto Rico, 1973–81. *J Natl Cancer Inst* **73**: 89–94

Biggar RJ, Curtis RE, Cote TR, Rabkin CS and Melbye M (1994) Risk of other cancers following Kaposi's sarcoma: relation to acquired immunodeficiency syndrome. *Am J Epidemiol* **139**: 362–368

Brousset P, Meggetto F, Attal M and Delsol G (1997) Kaposi's sarcoma-associated herpesvirus infection and multiple myeloma. *Science* **278**: 1972

Cesarman E, Chang Y, Moore PS, Said JW and Knowles DM (1995) Kaposi's sarcoma-associated herpesvirus-like DNA sequences in AIDS-related body-cavity-based lymphomas. *N Engl J Med* **332**: 1186–1191

Chang Y, Cesarman E, Pessin MS, Lee F, Culpepper J, Knowles D and Moore PS (1994) Identification of herpesvirus-like DNA sequences in AIDS-associated Kaposi's sarcoma. *Science* **266**: 1865–1869

Cook G, MacKenzie J, Sheldon G, Moran SG, Schulz TF, Jarret RF and Franklin IM (1997) Seroprevalence of Kaposi's sarcoma associated herpesvirus (KSHV/HHV8) in patients with multiple myeloma is similar to healthy controls. *Blood* **90**: 588a

Dupin N, Gorin I, Deleuze J, Agut H, Huraux J-M and Escande J-P (1995) Herpes-like DNA sequences, AIDS-related tumors, and Castleman's disease. *N Engl J Med* **333**: 798

Gao S-J, Kingsley L, Li M, Zheng W, Parravicini C, Ziegler J, Newton R, Rinaldo CR, Saah A, Phair J, Detels R, Chang Y and Moore PS (1996) KSHV antibodies among Americans, Italians and Ugandans with and without Kaposi's sarcoma. *Nature Med* **2**: 925–928

Geddes M, Franceschi S, Barchielli A, Falcini F, Carli S, Cocconi G, Conti E, Crosignani P, Gafà L, Giarelli L, Vercelli M and Zanetti R (1994) Kaposi's sarcoma in Italy before and after the AIDS epidemic. *Br J Cancer* **69**: 333–336

Grulich AE, Beral V and Swerdlow AJ (1992) Kaposi's sarcoma in England and Wales before the AIDS epidemic. *Br J Cancer* **66**: 1135–1137

Hjalgrim H, Melbye M, Pukkala E, Langmark F, Frisch M, Dictor M and Ekbom A (1996) Epidemiology of Kaposi's sarcoma in the Nordic countries prior to the AIDS epidemic. *Br J Cancer* **74**: 1499–1502

Hjalgrim H, Frisch M, Pukkala E, Tulinius H, Ekbom A, Dictor M, Langmark F, Hardarson S and Melbye M (1997) Risk of second cancers in classical Kaposi's sarcoma. *Int J Cancer* **73**: 840–843

Hjalgrim H, Tulinius H, Dalberg J, Hardarson S, Frisch M and Melbye M (1998) High incidence of classical Kaposi's sarcoma in Iceland and the Faroe Islands. *Br J Cancer* **77**: 1190–1193

Kaldor JM, Coates M, Vettom L and Taylor R (1994) Epidemiological characteristics of Kaposi's sarcoma prior to the AIDS epidemic. *Br J Cancer* **70**: 670–674

Levi F, Franceschi S and La Vecchia C (1993) Kaposi's sarcoma in the Swiss Canton of Vaud, 1974–90. *Eur J Cancer* **29a**: 1918–1919

Luppi M, Barozzi P, Marasca R, Ferrari MG and Torelli G (1997) Human herpesvirus 8 strain variability in clinical conditions other than Kaposi's sarcoma. *J Virol* **71**: 8082–8083

MacKenzie J, Sheldon J, Morgan G, Cook G, Schultz TF and Jarrett RF (1997) HHV-8 and multiple myeloma in the UK. *Lancet* **350**: 1144–1145

Marcelin AG, Dupin N, Bouscary D, Bossi P, Cacoub P, Ravaud P and Calvez V (1997) HHV-8 and multiple myeloma in France. *Lancet* **350**: 1144

Masood R, Zheng T, Tulpule A, Arora N, Chatlynne L, Handy M, Whitman Jr J, Kaplan M, Dosik M, Ablashi DV and Gill PS (1997) Kaposi's sarcoma-associated herpesvirus infection and multiple myeloma. *Science* **278**: 1970–1971

Muir CS, Waterhouse J, Mack T, Powell J and Whelan S (eds) (1987) *Cancer Incidence in Five Continents*, Vol 5, IARC scientific publication no. 88, IARC: Lyon

Parravicini C, Lauri E, Baldini L, Neri A, Poli F, Sirchia G, Moroni M, Galli M and Corbellino M (1997) Kaposi's sarcoma-associated herpesvirus infection and multiple myeloma. *Science* **278**: 1969–1970

Pastore C, Gloghini A, Volpe G, Nomdedeu J, Leonardo E, Mazza U, Saglio G, Carbone A and Gaidano G (1995) Distribution of Kaposi's sarcoma herpesvirus sequences among lymphoid malignancies in Italy and Spain. *Br J Haematol* **91**: 918–920

Rettig MB, Ma HJ, Vescio RA, Pold M, Schiller G, Belson D, Savage A, Nishikubo C, Wu C, Fraser J, Said JW and Berenson JR (1997*a*) Kaposi's sarcoma-associated herpesvirus infection of bone marrow dendritic cells from multiple myeloma patients. *Science* **276**: 1851–1854

Rettig MB, Vescio R, Moss T, Ma H, Schiller G and Berenson J (1997*b*) Detection of Kaposi's sarcoma-associated herpesvirus in the peripheral blood of multiple meyloma patients. *Blood* **90**: 587a

Said JW, Rettig MB, Heppner K, Vescio RA, Schiller G, Ma HJ, Belson D, Savage A, Shinkatu IP, Koeffler HP, Asou H, Pinkus G, Pinkus J, Schrage M, Green E and Berenson JR (1997) Localization of Kaposi's sarcoma associated herpesvirus in bone marrow biopsy samples from patients with multiple meyloma. *Blood* **90**: 4278–4282

Simpson GR, Schultz TF, Whitby D, Cook PM, Boshoff C, Rainbow L, Howard MR, Gao S-J, Bohenzky RA, Simmonds P, Lee C, de Ruiter A, Hatzakis A, Tedder RS, Weller IVD, Weiss RA and Moore PS (1996) Prevalence of Kaposi's sarcoma associated herpesvirus infection measured by antibodies to recombinant capsid protein and latent immunofluorescence antigen. *Lancet* **349**: 1133–1138

Tisdale JF, Stewart AK, Dickstein B, Dubé ID, Cappe D, Dunbar CE and Brown KE (1997) Human herpesvirus 8 in patients with multiple myeloma. *Blood* **90**: 588a

Whitby D, Boshoff C, Luppi M and Torelli G (1997) Kaposi's sarcoma-associated herpesvirus infection and multiple myeloma. *Science* **278**: 1971–1972

© Cancer Research Campaign 1998


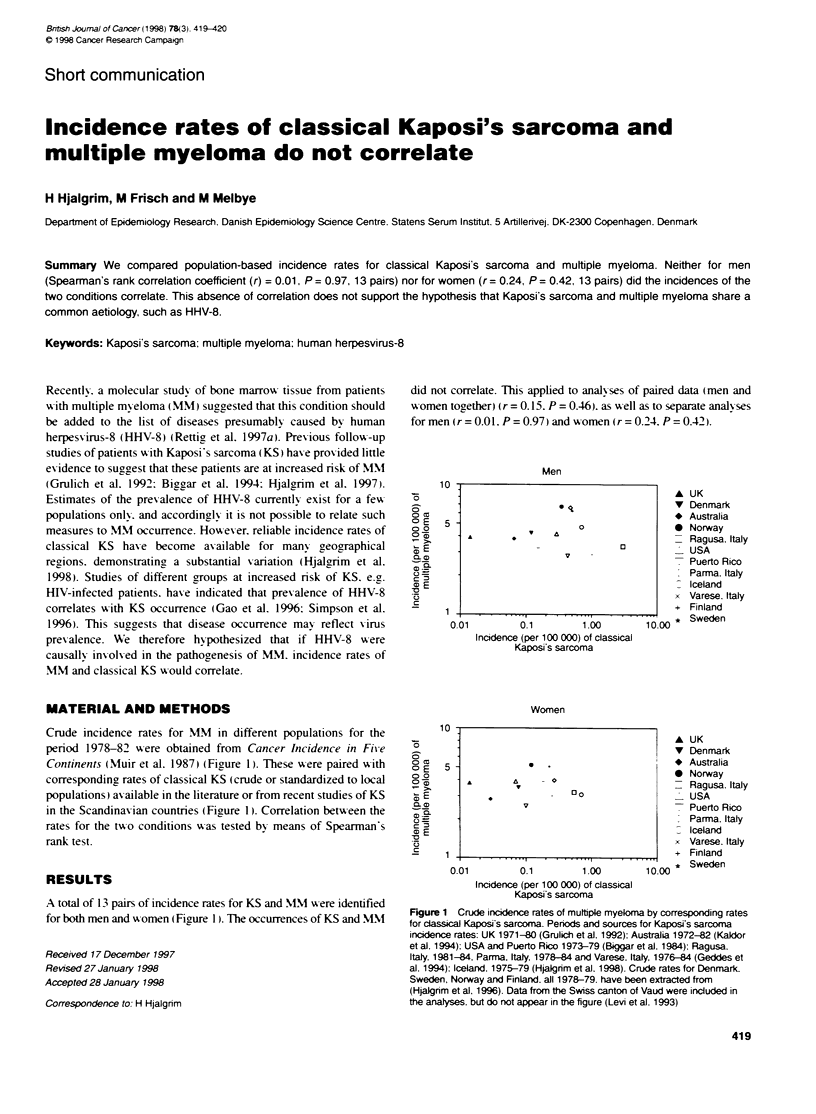

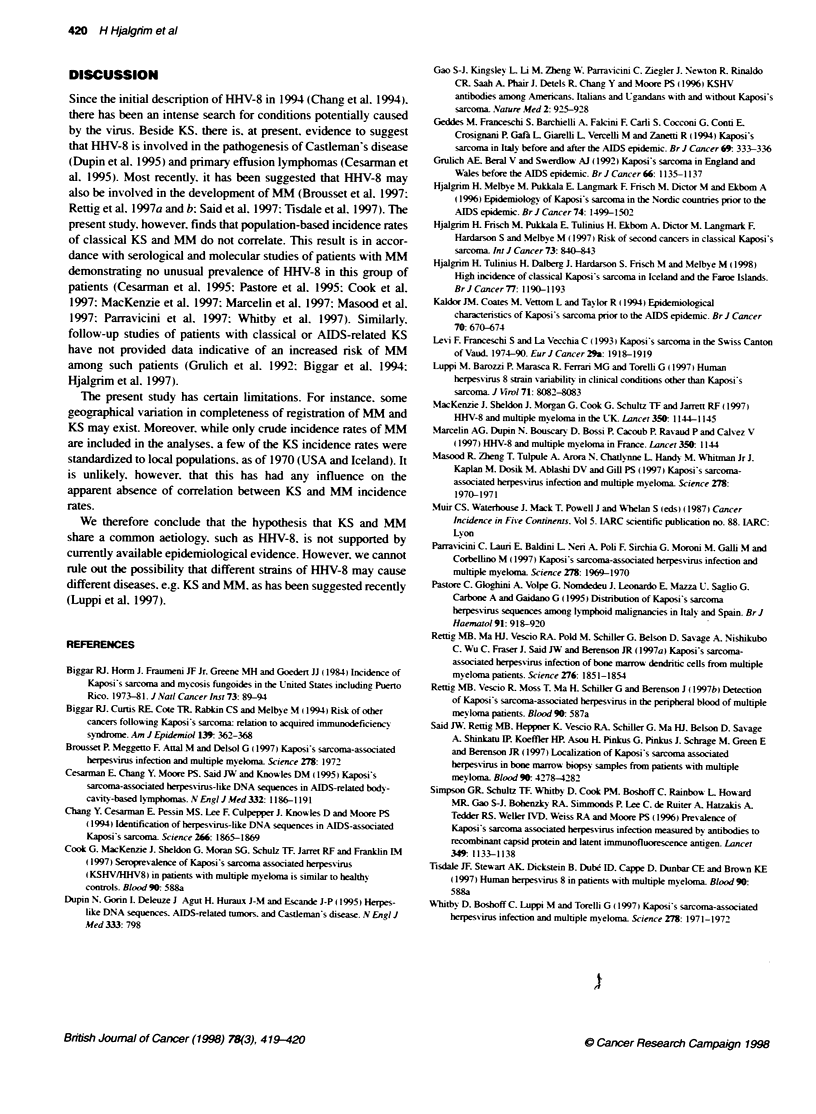


## References

[OCR_00252] Biggar R. J., Curtis R. E., Cote T. R., Rabkin C. S., Melbye M. (1994). Risk of other cancers following Kaposi's sarcoma: relation to acquired immunodeficiency syndrome.. Am J Epidemiol.

[OCR_00247] Biggar R. J., Horm J., Fraumeni J. F., Greene M. H., Goedert J. J. (1984). Incidence of Kaposi's sarcoma and mycosis fungoides in the United States including Puerto Rico, 1973-81.. J Natl Cancer Inst.

[OCR_00257] Brousset P., Meggetto F., Attal M., Delsol G. (1997). Kaposi's sarcoma-associated herpesvirus infection and multiple myeloma.. Science.

[OCR_00263] Cesarman E., Chang Y., Moore P. S., Said J. W., Knowles D. M. (1995). Kaposi's sarcoma-associated herpesvirus-like DNA sequences in AIDS-related body-cavity-based lymphomas.. N Engl J Med.

[OCR_00268] Chang Y., Cesarman E., Pessin M. S., Lee F., Culpepper J., Knowles D. M., Moore P. S. (1994). Identification of herpesvirus-like DNA sequences in AIDS-associated Kaposi's sarcoma.. Science.

[OCR_00278] Dupin N., Gorin I., Deleuze J., Agut H., Huraux J. M., Escande J. P. (1995). Herpes-like DNA sequences, AIDS-related tumors, and Castleman's disease.. N Engl J Med.

[OCR_00283] Gao S. J., Kingsley L., Li M., Zheng W., Parravicini C., Ziegler J., Newton R., Rinaldo C. R., Saah A., Phair J. (1996). KSHV antibodies among Americans, Italians and Ugandans with and without Kaposi's sarcoma.. Nat Med.

[OCR_00290] Geddes M., Franceschi S., Barchielli A., Falcini F., Carli S., Cocconi G., Conti E., Crosignani P., Gafà L., Giarelli L. (1994). Kaposi's sarcoma in Italy before and after the AIDS epidemic.. Br J Cancer.

[OCR_00295] Grulich A. E., Beral V., Swerdlow A. J. (1992). Kaposi's sarcoma in England and Wales before the AIDS epidemic.. Br J Cancer.

[OCR_00307] Hjalgrim H., Frisch M., Pukkala E., Tulinius H., Ekbom A., Dictor M., Langmark F., Hardarson S., Melbye M. (1997). Risk of second cancers in classical Kaposi's sarcoma.. Int J Cancer.

[OCR_00301] Hjalgrim H., Melbye M., Pukkala E., Langmark F., Frisch M., Dictor M., Ekbom A. (1996). Epidemiology of Kaposi's sarcoma in the Nordic countries before the AIDS epidemic.. Br J Cancer.

[OCR_00309] Hjalgrim H., Tulinius H., Dalberg J., Hardarson S., Frisch M., Melbye M. (1998). High incidence of classical Kaposi's sarcoma in Iceland and the Faroe Islands.. Br J Cancer.

[OCR_00314] Kaldor J. M., Coates M., Vettom L., Taylor R. (1994). Epidemiological characteristics of Kaposi's sarcoma prior to the AIDS epidemic.. Br J Cancer.

[OCR_00321] Levi F., Franceschi S., La Vecchia C. (1993). Kaposi's sarcoma in the Swiss Canton of Vaud, 1974-1990.. Eur J Cancer.

[OCR_00325] Luppi M., Barozzi P., Marasca R., Ferrari M. G., Torelli G. (1997). Human herpesvirus 8 strain variability in clinical conditions other than Kaposi's sarcoma.. J Virol.

[OCR_00328] MacKenzie J., Sheldon J., Morgan G., Cook G., Schulz T. F., Jarrett R. F. (1997). HHV-8 and multiple myeloma in the UK.. Lancet.

[OCR_00334] Marcelin A. G., Dupin N., Bouscary D., Bossi P., Cacoub P., Ravaud P., Calvez V. (1997). HHV-8 and multiple myeloma in France.. Lancet.

[OCR_00336] Masood R., Zheng T., Tupule A., Arora N., Chatlynne L., Handy M., Whitman J. (1997). Kaposi's sarcoma-associated herpesvirus infection and multiple myeloma.. Science.

[OCR_00349] Parravicini C., Lauri E., Baldini L., Neri A., Poli F., Sirchia G., Moroni M., Galli M., Corbellino M. (1997). Kaposi's sarcoma-associated herpesvirus infection and multiple myeloma.. Science.

[OCR_00354] Pastore C., Gloghini A., Volpe G., Nomdedeu J., Leonardo E., Mazza U., Saglio G., Carbone A., Gaidano G. (1995). Distribution of Kaposi's sarcoma herpesvirus sequences among lymphoid malignancies in Italy and Spain.. Br J Haematol.

[OCR_00363] Rettig M. B., Ma H. J., Vescio R. A., Põld M., Schiller G., Belson D., Savage A., Nishikubo C., Wu C., Fraser J. (1997). Kaposi's sarcoma-associated herpesvirus infection of bone marrow dendritic cells from multiple myeloma patients.. Science.

[OCR_00368] Said J. W., Rettig M. R., Heppner K., Vescio R. A., Schiller G., Ma H. J., Belson D., Savage A., Shintaku I. P., Koeffler H. P. (1997). Localization of Kaposi's sarcoma-associated herpesvirus in bone marrow biopsy samples from patients with multiple myeloma.. Blood.

[OCR_00379] Simpson G. R., Schulz T. F., Whitby D., Cook P. M., Boshoff C., Rainbow L., Howard M. R., Gao S. J., Bohenzky R. A., Simmonds P. (1996). Prevalence of Kaposi's sarcoma associated herpesvirus infection measured by antibodies to recombinant capsid protein and latent immunofluorescence antigen.. Lancet.

[OCR_00393] Whitby D., Boshoff C., Luppi M., Torelli G. (1997). Kaposi's sarcoma-associated herpesvirus infection and multiple myeloma.. Science.

